# Human Parathyroid Hormone Analog (3–34/29–34) promotes wound re-epithelialization through inducing keratinocyte migration and epithelial–mesenchymal transition via PTHR1-PI3K/AKT activation

**DOI:** 10.1186/s12964-023-01243-9

**Published:** 2023-08-23

**Authors:** Chunhao Zhou, Donghua Guan, Jialiang Guo, Shangbo Niu, Zhihai Cai, Chengfu Li, Chenghe Qin, Wenjuan Yan, Dehong Yang

**Affiliations:** 1 Department of Orthopaedics, Nanfang Hospital, Division of Spine Surgery, Southern Medical University, 1838 North Guangzhou Ave, Guangzhou, 510515 P. R. China; 2 Department of Emergency, Zengcheng Branch of Nanfang Hospital, Southern Medical University, No. 28 Chuangxin Avenue Yongning Street, Guangzhou, 511340 P. R. China; 3 Department of Orthopaedics, Nanfang Hospital, Division of Orthopaedic Trauma, Southern Medical University, 1838 North Guangzhou Ave, Guangzhou, 510515 P. R. China; 4 Department of Stomatology, Nanfang Hospital, Southern Medical University, 1838 North Guangzhou Ave, Guangzhou, 510515 P. R. China

**Keywords:** Wound healing, Re-epithelialization, PI3K/AKT, Cell migration, Epithelial-mesenchymal transition, hPTH (3–34/29–34)

## Abstract

**Background:**

Re-epithelialization is important in the process of wound healing. Various methods have been identified to expedite the process, but their clinical application remains limited. While parathyroid hormone (PTH) has shown promising results in wound healing due to its role in promoting collagen deposition and cell migration, application is limited by its potentially inhibitive effects when being continuously and locally administrated. Herein, we developed a novel PTH analog, Human parathyroid hormone (hPTH) (3–34/29–34) (henceforth MY-1), by partially replacing and repeating the amino acid sequences of hPTH (1–34), and evaluated its effect on skin wound re-epithelialization.

**Methods:**

CCK-8, colony formation unit assay, and Ki67 immunofluorescent staining were performed to evaluate the effect of MY-1 on HaCaT cell proliferation. Then, wound scratch assay, Transwell assay and lamellipodia staining were carried out to evaluate the effect of MY-1 on cell migration. Moreover, the epithelial–mesenchymal transition (EMT) markers were measured using qPCR and western blot analysis. For in-vivo drug delivery, gelatin methacryloyl (GelMA) hydrogel was employed to load the MY-1, with the physicochemical characteristics evaluated prior to its application in wound models. Then, MY-1’s role in wound healing was determined via acute skin wound models. Finally, the mechanism that MY-1 activated was also detected on HaCaT cells and in-vivo wound models.

**Results:**

In-vitro, MY-1 accelerated the migration and EMT of HaCaT cells, while having little effect on cell proliferation. GelMA and MY-1-incorporated GelMA hydrogels showed similar physicochemical characteristics and were used in the in-vivo studies, where the results revealed that MY-1 led to a stronger re-epithelialization by inducing basal keratinocyte migration and EMT. Further studies on in-vivo wound models and in-vitro HaCaT cells revealed that MY-1 regulated cell migration and EMT through activating PI3K/AKT signaling. The parathyroid hormone type 1 receptor (PTHR1), the main receptor of PTH, was found to be the upstream of PI3K/AKT signaling, through interfering PTHR1 expression with a small interference RNA following detection of the PI3K/AKT activation.

**Conclusion:**

Collectively, our study demonstrated that MY-1 accelerates skin wound re-epithelialization by inducing keratinocyte migration and EMT via PTHR1-PI3K/AKT axis activation.

Video Abstract

**Supplementary Information:**

The online version contains supplementary material available at 10.1186/s12964-023-01243-9.

## Background

Skin wound healing represents a highly orchestrated process that is regulated by numerous factors and comprised of three overlapping phases: inflammation, tissue regeneration, and remodeling [1, 2]. The re-epithelialization of the epidermis, which involves the migration and proliferation of basal keratinocytes to cover the wound area, plays an indispensable role in the wound regeneration phase by contributing to the recovery of skin integrity and the restoration of its protective functions [3]. Normally, this process is initiated at the wound margins where sedentary keratinocytes become activated and migrate toward the wound center in order to restore the intact skin coverage [4]. Although the process of epidermis repair has been well described, the successful re-establishment of intact epidermis coverage remains a challenge. Complicating factors such as infection, diabetes, malnutrition, and immune disorders impede re-epithelialization and ultimately result in delayed and non-healing wounds [5]. Chronic wounds not only cause physical and psychological distress to individuals, but also impose a significant economic burden. It was reported that over 40 million people are affected by chronic wounds, while nearly 2% of citizens in developing nations will experience a chronic wound during their lifetime [6, 7]. Hence, the pursuit of novel treatment modalities aimed at accelerating wound healing and diminishing the incidence of chronic wounds represents a research topic of considerable interest.

Due to the unique role of re-epithelialization, understanding its mechanism and exploring the directional treatment strategies would offer significant benefits to wound healing. Literature has verified that both the migration and proliferation of keratinocytes comprise a substantial component of the epidermis re-epithelialization process, through which wound closure was significantly accelerated [8, 9]. Besides the increased number of keratinocytes, epithelial–mesenchymal transition (EMT) represents an important process by which stationary keratinocytes experience cytoskeleton re-arrangements and morphology transformation to become endowed with special features that fit their functional destination. Stimulated by increased growth factors, keratinocytes around the wound area initiate partial EMT through losing epithelial phenotypes of cell polarity and cell–cell adhesion, and downregulating the expression of E-cadherin (E-cad), while possessing mesenchymal phenotypes including enhanced migratory abilities, fibroblastic morphology, and the expression of Vimentin (Vim) and N-cadherin (N-cad) [10, 11]. To date, considerable evidence has verified that the EMT of keratinocytes is closely correlated to the motility of keratinocytes and directedly influences the re-epithelialization process [2]. A considerable number of drugs, growth factors, and cytokines have been identified as potential reagents to enhance the EMT. In terms of the most frequently reported reagents, transforming growth factor beta (TGF-β) and epidermal growth factor were confirmed to strongly induce the EMT and cell migration during re-epithelialization [12, 13]. The other previously reported potential candidates include fibroblast growth factor [14], tumor necrosis factor-alpha [15], and calcipotriol [16]. However, while the positive effects have been revealed in experiments, the use of these regents for wound managing has not been widely explored in clinical practice. Certain definite or potential limitations such as a short half-life, unstable activity, complicated regulatory mechanisms, and undesirable or even harmful effects, inevitably hinder their clinical administration.

The parathyroid hormone (PTH) as a physiological hormone secreted by the parathyroid gland vitally regulates the balance of calcium and phosphorus [17]. hPTH (1–34) (teriparatide), a truncated PTH peptide, was widely applied in the treatment of osteoporosis [18, 19]. PTH and related proteins (PTHrP) also exert effects on angiogenesis and the deposition of extracellular matrix [6, 20, 21]. Meanwhile, the positive effect of PTH and analogs on cell migration and/or EMT was studied in a diversity of cells, which includes mesenchymal stem cells [22], vascular endothelial cells [23], intestinal epithelial cells [24], renal tubular epithelial cells [25], and myoblasts [26]. However, the relationship between PTH and skin wound healing remains unclear, only a few studies reported the application of PTH and its derivative accelerated the process of re-epithelialization [6, 20]. Considering the negative effects of PTHs on bone metabolism when they are continuously and locally administrated, the local application of PTH might lead to inhibitive effects on wound healing.

In our previous study, a novel PTH analog, hPTH (3–34/29–34) (MY-1) was designed [27]. Previous exploration on a rat calvarial bone defect model verified that the local application of MY-1 accelerated bone repair via collagen deposition and cell migration, which confirmed MY-1’s positive role when administrated locally and continuously [28]. Moreover, in a former study on a rat skin defect model, we found that an MY-1-loaded hydrogel promoted wound healing. However, the mechanism of MY-1 on wound healing remained unknown. In the present investigation, MY-1 was locally delivered on acute cutaneous defects with a gelatin methacryloyl (GelMA) hydrogel as the scaffold carrier, and we focused on the process of re-epithelialization to explore the effects of MY-1 on re-epithelialization. Additionally, in-vivo and in-vitro studies were carried out to investigate the underlying mechanisms for such a novel phenomenon.

## Methods and materials

### Synthesis and storage of MY-1 peptide

MY-1 was synthesized by Mecklin, Co., Ltd (Shanghai, China). The peptide powder was dissolved to a concentration of 1 mM with distilled water containing 0.1% trifluoroacetic acid (Mecklin) and stored at -20 ℃ for further usage.

### Cell culture

The human immortalized keratinocyte cell line (HaCaT) was purchased from the Cell Bank of the Chinese Academy of Sciences (Shanghai, China). The cells were cultured with Dulbecco’s modified Eagle’s medium (Gibco, Rockville, MD, USA) containing 10% fetal bovine serum ([FBS]; Gibco) and 1% penicillin and streptomycin (Gibco) in a cell culture condition of 5% CO_2_ and 95% humidity at 37 ℃. The culture medium was changed every 2 days and the cells were passaged once confluence had been reached.

### CCK-8 cell counting assay

HaCaT cells were plated in 96-well plates (2 × 10^3^ cells per well) and subjected to various concentrations of MY-1 peptide from 1 nM to 10 μM. At the timepoints of 24 h and 72 h, the complete medium was replaced with 100 μL fresh DMEM (Gibco) containing 10 μL CCK-8 solution (Dojindo, Kumamoto, Japan) and incubated at 37℃ for 2 h. The optical density value at the wavelength of 450 nm was measured via SpectraMax i3x multi-mode microplate reader (Molecular Devices Co., Ltd, San Jose, CA, USA).

### Colony formation unit assay

Suspended HaCaT cells were seeded in a 6-well plate (1000 cells per well) and cultured for 24 h to ensure complete adhesion. Then, the HaCaT cells were washed three times with phosphate buffered saline (PBS) and incubated in fresh medium with graded concentrations of MY-1 (0 nM, 10 nM, and 100 nM). After 7 days of incubation, the clones were fixed with 4% paraformaldehyde and stained with 0.1% crystal violet for 15 min. Colonies containing 50 or more cells were selected and quantified by Image J software.

### Immunofluorescence staining of HaCaT cells

HaCaT cells were seeded (10^4^ cells/mL) in confocal dishes and different treatments were applied for 48 h. Then, the HaCaT cells were fixed with 4% paraformaldehyde for 15 min at room temperature, followed by permeabilizing with 0.5% (v/v) Triton X-100 (Solarbio, Beijing, China) for 15 min. After washing with PBS three times, the cells were blocked in 5% bovine serum albumin (BSA, Solarbio) for 1 h and incubated in primary antibodies overnight at 4 ℃. Subsequently, the cells were washed again and incubated in Alexa Fluor 488- or 594-conjugated secondary antibodies (Bioss, Beijing, China) for 2 h at room temperature. Finally, 4′,6-diamidino-2-phenylindole (DAPI, Beyotime, Shanghai, China) was utilized to stain the nuclei for 10 min. Fluorescence images were observed by confocal laser scanning microscopy (LSM-980; Carl Zeiss, Oberkochen, Germany). The primary antibodies used in this study included primary antibodies against Ki67 (1:1000; Affinity Biosciences, Cincinnati, OH, USA), Vim (1:1000; Abcam, Cambridge, UK), E-cad (1:1000; Affinity Biosciences), p-AKT (1:1000; Affinity Biosciences), and PTHR1 (1:1000; Affinity Biosciences).

### Scratch wound healing assay

HaCaT cells (4.5 × 10^5^per well) were seeded in a 12-well plate until 90% confluency was reached. Then, the cells in different wells were grouped and subjected to different treatments for 24 h. When the cells reached 100% confluency, scratch wounds were created using sterile yellow pipette tips and the cell fragments were removed with PBS wash buffer. Next, pictures of different groups were captured at 0 h. The cells in each group were continuously cultured with different mediums for 24 h and the remaining wound areas were captured and measured by Image J software.

### Transwell assay

For the Transwell assay, HaCaT cells were pre-treated with different concentrations of MY-1 for 24 h. Then, the cells were resuspended in a serum-free medium and adjusted to a concentration of 5 × 10^5^ cells/ml. Thereafter, 100 μL of suspended HaCaT cells was loaded in the upper chamber of a 24-well, 8 um pore-size Transwell plate (Corning, NY, USA), while a culture medium containing 2% FBS with different mediums was added into the lower chamber. After 48 h of incubation, the cells in the upper chamber were fixed with 4% paraformaldehyde and unmigrated cells were swabbed with cotton swabs. The migrated cells were stained with 0.1% (w/v) crystal violet (Leagene, Beijing, China) for 15 min and the images photographed by IX73 inverted phase-contrast microscope (Olympus, Tokyo, Japan).

### Cytoskeleton (F-actin) staining

For cytoskeleton and lamellipodia staining, HaCaT cells were seeded in the confocal dishes at a density of 1 × 10^4^ per well. After adhesion, the complete medium was exchanged with fresh medium (featuring 2% FBS) containing different agents, and incubated for 12 h. Then, HaCaT cells in each group were fixed with 4% paraformaldehyde for 15 min, washed with PBS three times, and stained with rhodamine-labeled phalloidin for 30 min, followed by 10 min of DAPI staining. The cell morphology and lamellipodia were observed using the LSM-980 confocal microscope (Zeiss).

### Cell morphology

HaCaT cells (2 × 10^4^ per well) were seeded in a 12-well plate and cultured for 24 h. Then, the medium was replaced with DMEM containing 2% FBS and the cells were subjected to graded concentrations of MY-1 for 5 days. The morphology of the HaCaT cells in each group was captured using an inverted phase-contrast microscope (IX73; Olympus).

### Quantitative real-time PCR analysis

Prior to qPCR analysis, the HaCaT cells were seeded in a 6-well plate and subjected to graded concentrations of MY-1 (0 nM, 10 nM, and 100 nM) for 24 h. The total RNA was extracted with an RNA purification kit (Vazyme, Nanjing, China) and reverse transcription was performed with a cDNA reverse transcription kit (Vazyme). qPCR was performed employing SYBR Green qPCR Master Mix (Vazyme) and the expression levels of relative genes were calculated utilizing the 2^−ΔΔCT^ method, with GAPDH used as a reference for normalization. The qPCR primers provided by Qingke (Beijing, China) are listed in Table 1.

**Table 1:** Sequences of primers used for the quantitative reverse transcriptase-polymerase chain reaction (qRT-PCR)

Gene name	Forward primer sequences	Reverse primer sequences
**E-cadherin**	ACATACACTCTCTTCTCTC	GTCATTCTGATCGGTTAC
**N-cadherin**	ATCATCCTGCTTATCCTT	TTATCTCTTACATCATCTTCTG
**Vimentin**	AACCTGAGGGAAACTAAT	TTGATAACCTGTCCATCT
**Snail1**	CGCTCTTTCCTCGTCAGG	TGGAAGGTAAACTCTGGATTAGA
**GAPDH**	GGTAGTGGCGATGGCGG	CTGTAGGAACGCCGACTGC

### Western blot analysis

Whole-cell protein was extracted with the RIPA lysis buffer containing protease inhibitor (Beyotime) and protein phosphatase inhibitor (Beyotime) on ice. Equal amounts of protein (30 μg) were loaded into 10% (w/v) SDS-PAGE and transferred to a polyvinylidene difluoride membrane (Millipore, St. Louis, MO, USA), with the membrane then incubated with primary antibodies at 4 °C overnight, followed by three series of TBST washing and 2 h of HRP-conjugated secondary antibodies incubation at room temperature. The proteins were visualized through chemiluminescence and imaged on a Gelview 6000 Pro (BLT Co., Ltd, Guangzhou, China). The protein bands were quantified by densitometry analysis using Image J software.

The primary antibodies used in this study included antibodies against GAPDH (1:1000; Affinity Biosciences), Vim (1:1000; Abcam), E-cad (1:1000; Affinity Biosciences), N-cad (1:1000; Santa Cruz, Cincinnati, OH, USA), PI3K (1:1000; Santa Cruz), AKT (1:1000; Santa Cruz), p-PI3K (1:1000; Affinity Biosciences), p-AKT (1:1000; Affinity Biosciences), and PTHR1 (1:1000; Affinity Biosciences).

### Immunofluorescence staining of HaCaT cells

HaCaT cells were fixed with 4% paraformaldehyde for 15 min, permeabilized with a 0.5% (v/v) Triton X-100 in PBS for 15 min, and blocked in 5% BSA (Solarbio) for 1 h. Then, HaCaT cells were incubated in primary antibodies overnight at 4 ℃. Subsequently, the cells were incubated in Alexa Fluor 488 and/or 594-conjugated secondary antibodies (1:1000; Abcam) at room temperature for 2 h. Finally, DAPI staining solution (Beyotime) was employed to stain the nuclei of the cells. Images were collected by LSM980 confocal laser scanning microscopy (Carl Zeiss). The primary antibodies utilized in immunofluorescence (IF) staining included anti-Vim (1:400), E-cad (1:250), Ki67 (1:250), and p-Akt (1:250).

### siRNA transfection

siPTHR1 and a negative control were designed and synthesized by Qingke Co., Ltd. (Beijing, China). siRNA transfection was carried out when the HaCaT cells reached 80% confluence using Lipo3000 transfection reagent (Invitrogen, Carlsbad, CA, USA) according to the manufacturer’s instructions.

### In-vivo experiments

#### Preparation of GelMA hydrogels

The 30% methacrylation degree GelMA used in this study was purchased from the Engineering for Life Co., Ltd (Suzhou, China), with the 5% (w/v) GelMA hydrogels prepared according to the manufacturer’s instructions. The dissolved GelMA was filtered immediately using a 0.22 μm filter (Millipore, Shanghai, China) and stored at 4 ℃ in darkness for further experiments.

#### Morphology and structural characterization of hydrogels

The morphologies of GelMA and GelMA-MY-1 were observed with a field emission scanning electronic microscope (SEM, Sigma 300; Carl Zeiss). Energy dispersive spectrometry (EDS) analysis was subsequently carried out to assess the elemental composition of the two types of hydrogels. The chemical composition of GelMA and GelMA-MY-1 was characterized by Fourier transform-infrared spectroscopy (Vector 33; Bruker, Berlin, Germany).

#### Swelling and degradation assays

To detect the swelling character of the hydrogels, the GelMA and GelMA-MY-1 were lyophilized and weighed as M(0). The hydrogels were immersed in PBS and placed in an incubator at 37℃. At the timepoints of 1, 3, 6, 12, 24, and 36 h, the water outside of the hydrogels was removed and the weights of the swelling hydrogels were measured as M(1). The swelling ratio of each timepoint was calculated employing the equation: swelling ratio = (M(l)-M(0))/M(0).

For the degradation assay, the hydrogels were lyophilized and the initial weights recorded as W0. Then, the hydrogels were immersed in PBS and placed in an incubator at 37℃. The hydrogel samples were removed at different timepoints and lyophilized to determine the remaining weights (W1). The degradation rate of each timepoint was calculated utilizing the equation: degradation ratio = (W(l)-W(0))/W(0).

#### Peptide distribution and hydrogel drug release

To detect the distribution of MY-1 in the hydrogels, fluorescein isothiocyanate (FITC)-labeled MY-1 mixed with GelMA was observed with the LSM-980 confocal microscope (Carl Zeiss), and then a 3D image was reconstructed. For the in-vitro peptide release assay, a standard curve was generated to correlate the optical density value of FITC with graded concentrations of MY-1-FITC. Then, GelMA-MY-1-FITC was cross-linked, immersed in PBS in centrifuge tubes, and placed in a shaker at 37 °C in a dark location. The 100 µL supernatants were collected at a certain timepoint, and an equal volume of PBS was added to the system. The optical density values of the supernatants were measured at the excitation wavelength of 488 nm, and were subsequently entered into the formula of standard curve to calculate the concentrations of MY-1 at each timepoint.

### In-vivo wound model

The animal experiments were approved by the Animal Ethics Committee of Nanfang Hospital of Southern Medical University (Grant No. NFYY-2022–0417). Male SD rats (SPF, 250 ± 30 g) were purchased from the Animal Center of Southern Medical University and housed under standard temperature and humidity conditions. Surgery was carried out after anesthetizing by the inhalation of isoflurane. Two symmetric full-thickness skin wounds (diameter: 15 mm) were created on the back of the rats. Then, the surgical-treated rats were randomly divided into three groups: (1) the Blank group (50 μL PBS per wound), (2) the GelMA group (50 μL GelMA per wound), and (3) the GelMA-MY-1 group (50 μL GelMA containing 0.1 μg MY-1 per wound). To investigate whether the PI3K/AKT signaling pathway was involved in the MY-1-induced in-vivo wound healing process, the rats were randomly divided into the following groups: (1) the GelMA group (50 μL GelMA per wound), (2) the GelMA-MY-1 group (50 μL GelMA + 0.1 μg MY-1 per wound), (3) the GelMA-MY-1-LY294002 (LY, PI3K inhibitor; Beyotime) group (50 μL GelMA + 0.1 μg MY-1 and 50 μg LY per wound), and (4) the GelMA-LY group (50 μL GelMA + 50 μg LY per wound). After different treatments, the wounds were covered with Tegaderm™ films (3 M, St. Paul, MN, USA) and protected with gauzes and bandages. The wounds were photographed at days 0, 3, 7, 10, or 14 post-surgery and the healing effects were evaluated with Image J software. The rats from each group were humanely sacrificed at day 7 and day 14 post-surgery, and the samples were harvested and fixed in 4% paraformaldehyde.

### Histology staining

For immunohistochemical staining, 4-μm-thick sections received antigen retrieval and endogenous peroxidase activity blocking. Then, the samples were incubated in goat serum for 30 min at room temperature to block the nonspecific antigen, followed by incubating in primary antibodies against K10 (1:200; Santa Cruz), K14 (1:200; Santa Cruz), and p-AKT (1:250; Affinity Biosciences) overnight at 4 ℃. Finally, the immune reactivities of the sections were determined using the HRP-streptavidin detection system (ZSGB-bio, Beijing, China). Images from above-mentioned sections were acquired with a BX63 fluorescence microscope (Olympus).

For immunofluorescence staining, 4-μm-thick sections received antigen retrieval and permeabilizing with 0.5% Triton X-100. After blocking with goat serum (Solarbio), primary antibodies against Ki67 (1:250; Affinity Biosciences), Cytokeratin (Pan) (1:200; Huabio, Hangzhou, China), E-cad (1:200; Affinity Biosciences), Vim (1:500; Abcam), and N-cad (1:200; Santa Cruz) were loaded and kept overnight at 4 ℃. Then, Alexa Fluor 488- and Alexa Fluor 594-conjugated secondary antibodies (Abcam) were loaded at room temperature for 2 h. The nuclei were stained with DAPI (Beyotime), with the images visualized via the LSM-980 confocal microscope (Carl Zeiss).

### Statistical analysis

All data are expressed as mean ± SD from at least three experimental repeats. The unpaired Student’s *t* test or Mann–Whitney U test was used for comparison between two groups with/without a normal distribution. One-way analysis of variance (ANOVA) with Tukey’s post hoc test was utilized for multiple comparisons to determine the significance of difference. All the data were processed using SPSS (v.22) (SPSS Inc., Chicago, IL, USA). A value of *p* < 0.05 was considered statistically significant.

## Results

### MY-1 has no effect on HaCaT cell proliferation but promotes cell migration

**Fig. 1:**
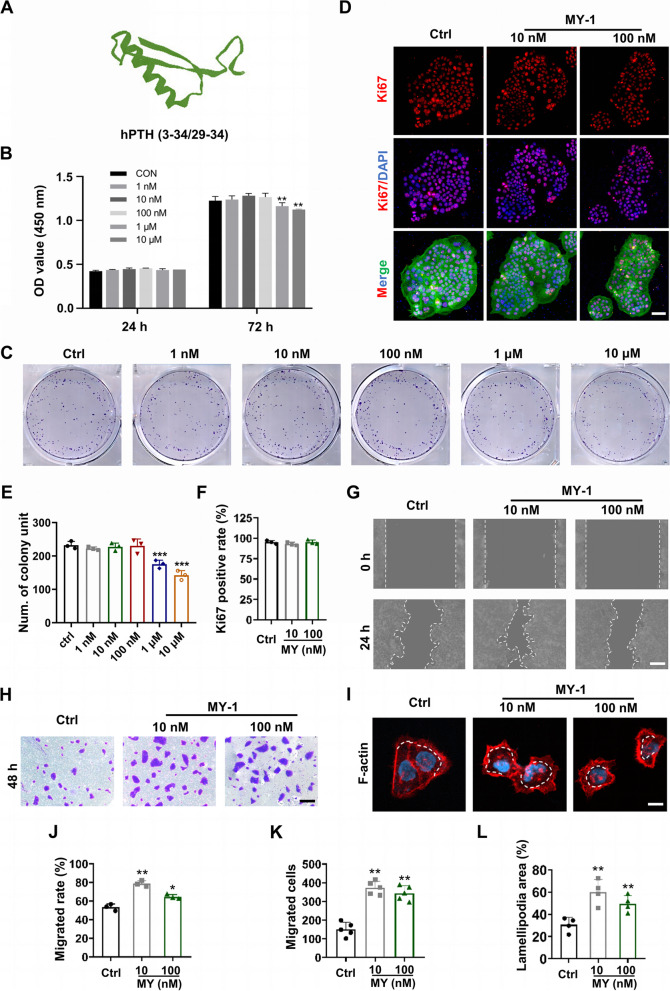
MY-1 had little effect on HaCaT cell proliferation but promoted migration. **A** The tertiary structure of MY-1 predicted by AlphaFold2. **B** Cell proliferation measured by CCK8 kit after being subjected to graded concentrations of MY-1 (0–10 μM) for 24 h and 72 h. **C** and **E** Cell proliferation measured by Ki67 immunofluorescent staining after incubation withMY-1 (0,10, and 100 nM) for 48 h (**C**). The ratios of Ki67 + cells quantified and compared (**E**). **D** and** F** Representative images of the CFU of HaCaT cells after their treatment with PBS or MY-1 for 7 days (**D**). The quantified number of colony units (**F**). **G** and** J** Wound scratching images captured at 0 h and 24 h after incubating with/without MY-1 (**G**), the measured and analyzed ratios of migrated areas (**J**). **I** and** K** Images of transwell assay on HaCaT cells after incubation with/without MY-1 for 48 h (**I**). The migrated cells counted respectively (**K**). **H** and** L** Representative images of cell scattering assay (**H**) and quantitative analysis (**L**) of the ratio of lamellipodia areas versus total cell areas. The data represent the mean ± SD. *, *p* < 0.05; **, *p* < 0.01; ***, *p* < 0.001 vs control group. Scale bar: 50 um (in **C**), 100 μm (in **G** and **I**), 25 μm (in **H**). Abbreviation: Ctrl, Control

Prior to evaluating the effects of MY-1 on cells, the MY-1 structure was first predicted by AlphaFold2. As per the results, MY-1 is a novel short peptide with a simple structure containing several classic α-helices, as well as a β-turn and a random coil (Fig. 1A). To examine MY-1’s effect on the cell proliferation, HaCaT cells were seeded in a 96-well plate and treated with MY-1 at graded concentrations (0.1–10 μM) for 24 h or 72 h. Then, the cell activities in each group were measured utilizing a CCK-8 kit. As per the results, incubation with lower concentrations of MY-1 (10 nM or 100 nM) exhibited little effect on the cell proliferation, while higher MY-1 concentrations (1 μM and 10 μM) even showed an inhibitive effect on the cell viability (Fig. 1B). Next, to confirm the results of the CCK-8 assay, colony formation unit (CFU) assays for cell proliferation evaluation were carried out. In line with the CCK-8 assay, the null ability of MY-1 on cell proliferation was validated by CFU assay, with similar numbers of HaCaT cell colony units in the three groups (Fig. 1C and E). Based on these results, 10 nM and 100 nM MY-1 were selected for the following studies. Besides CCK-8 and CFU assays, Ki67, a classic marker for cell proliferation, was also detected in the HaCaT cells via immunofluorescent staining. Through evaluating the ratio of Ki67 + cells in the three groups, Ki67 immunofluorescent staining exhibited a similar result to the above-mentioned assays, confirming MY-1’s limited effect on cell proliferation (Fig. 1D and F).

Since MY-1 had exhibited little effect on the cell proliferation, its role in cell migration was next evaluated. The results of the wound scratching assay indicated that the incubation of MY-1 enhanced cell migration, especially when the HaCaT cells were subjected to 10 nM MY-1 (Fig. 1G and J). Similarly, MY-1’s role in cell migration was confirmed by transwell assay, in which the MY-1-treated groups exhibited increased numbers of migrated cells following 48 h of incubation (Fig. 1H and K). Since the cytoskeleton reconstruction and lamellipodia formation are indispensable stages for motility acquisition, measuring the areas of lamellipodia at the same timepoint represents another classic assay for single-cell migration. Using cell scattering assay, we subsequently compared the capacity of the cell migration in the three groups through measuring the areas of lamellipodia. The results showed that MY-1 induced greater lamellipodia extension in the cell periphery in the HaCaT cells, which confirmed the positive role of MY-1 in cell migration (Fig. 1I and L).

### MY-1 induced partial EMT in HaCaT cells

**Fig. 2:**
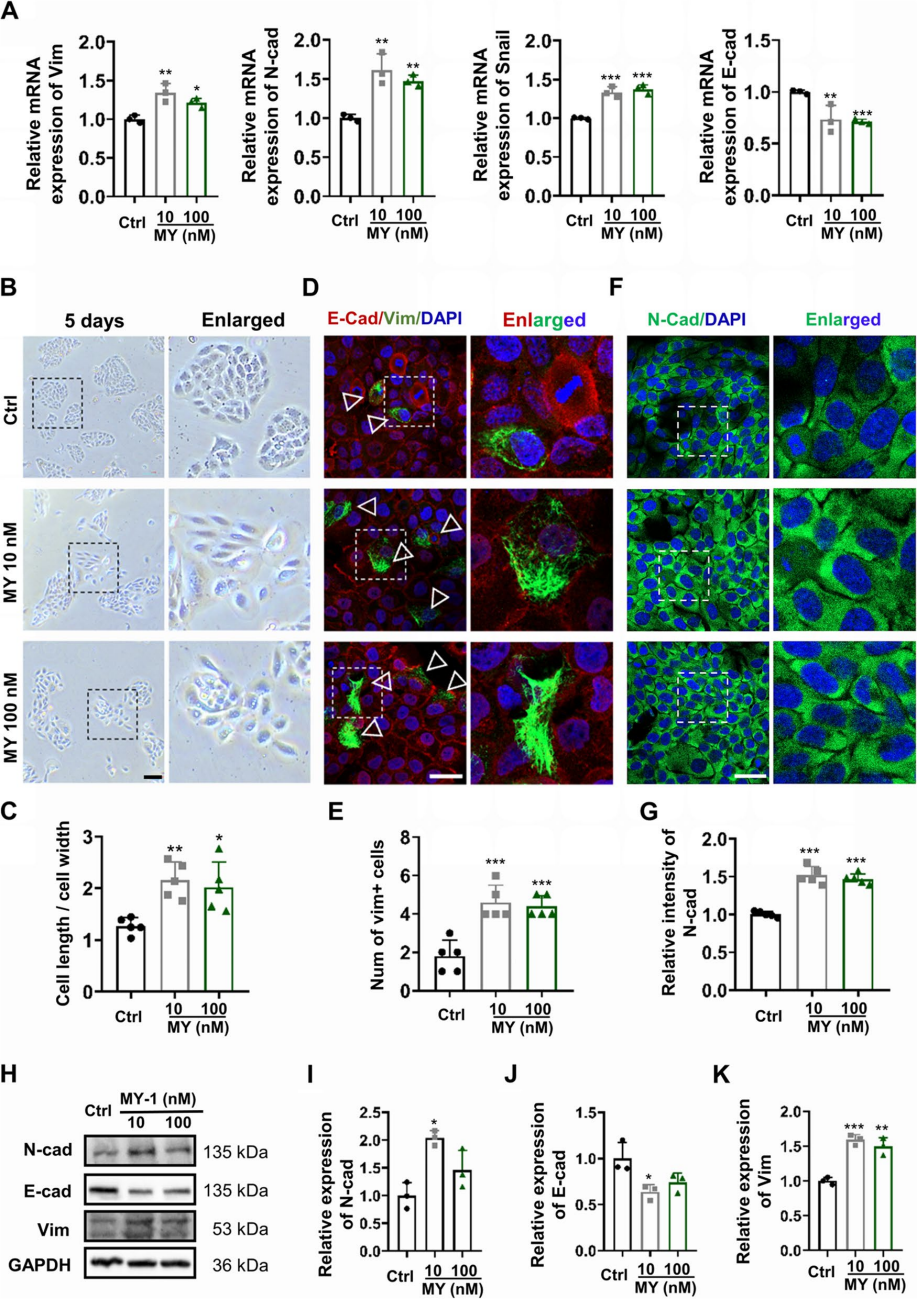
MY-1 induced EMT of HaCaT cells. **A** Relative mRNA expression levels of Vim, N-cad, Snail, and E-cad on HaCaT cells after being subjected to MY-1 (0, 10, and 100 nM) for 24 h. **B** and **C** Images of cell morphology after incubating in a low serum medium (2% FBS) containing MY-1 for 5 days (**B**). The ratio of the length/width of cells (**C**). **D** and **E** Immunofluorescent staining of E-cad and Vim on HaCaT cells cultured with MY-1 for 48 h (**D**), and the numbers of Vim + cells (**E**). **F** and **G** Immunofluorescent staining of N-cad on HaCaT cells cultured with MY-1 for 48 h (**F**). The intensity of N-cad (**G**). **H–K** The expression of EMT markers (E-cad, Vim, and N-cad) in HaCaT cells after treatment with MY-1 and evaluation by western blot assay (**H**). The intensity of each band measured and normalized to GAPDH, and then calculated as the ratio of the controls (**I**–**K**). The data represent mean ± SD. *, *p* < 0.05; **, *p* < 0.01; ***, *p* < 0.001 vs control group. Scale bar: 100 μm (in **B**), 50 μm (in **D** and **F**). Abbreviation: Ctrl, Control; E-cad, E-cadherin; Vim, Vimentin; N-cad, N-cadherin

EMT is a crucial process that affects keratinocyte migration, during which epithelial cells experienced morphology changes to commence the migratory phenotype [29]. As MY-1 exhibits a strong effect on regulating keratinocyte migration, we next evaluated its role in the EMT of keratinocytes. After incubation with MY-1 for 24 h, qPCR showed that the expression of EMT markers (Vim and N-cad) was upregulated after co-culture with MY-1 (Fig. 2A). Since EMT was closely relevant to the cell shape re-construction, the shape changes of the HaCaT cells in response to MY-1 treatment were then assessed. After 5 days of MY-1 incubation, the shape of the HaCaT cells was widely spread and the density was decreased, thus indicating that the characteristic of the HaCaT cells had changed (Fig. 2B and C). Subsequently, to further verify the effect of MY-1 on EMT, the protein of the above-mentioned EMT markers was evaluated with immunofluorescence staining and western blot analysis. As per the results, the immunofluorescence staining revealed that the application of MY-1 decreased the expression level of E-cad while notably increasing the expression of mesenchymal markers such as Vim and N-cad (Fig. 2D–G). Consistent with the results of immunofluorescence staining, the expression changes of EMT markers in the HaCaT cells were also detected and confirmed in the western blot analysis (Fig. 2H–K). Collectively, the in-vitro administration of MY-1 led to the EMT of keratinocytes.

### Characterizations of the GelMA and GelMA-MY-1 hydrogels

**Fig. 3:**
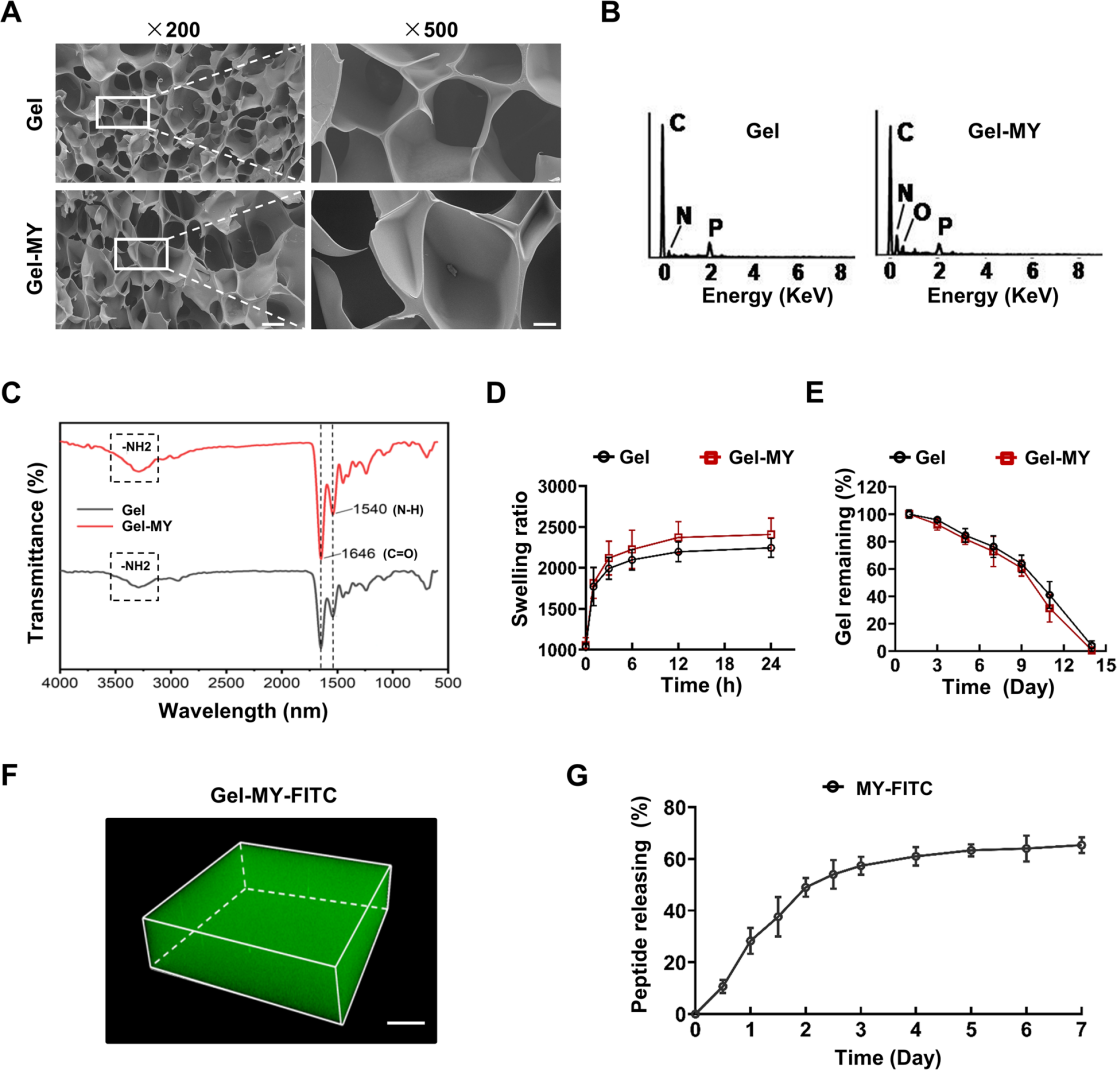
Characterization of GelMA and GelMA-MY-1 hydrogels. **A** SEM images of GelMA and GelMA-MY-1 hydrogels. **B** EDS spectra of GelMA and GelMA-MY-1 hydrogels. **C** FTIR spectra of GelMA and GelMA-MY-1 hydrogels. **D** In-vitro swelling quantified by the weight of hydrogels over its freeze-dried weight when the hydrogels were maintained in PBS at 37℃. **E** GelMA and GelMA-MY-1 hydrogels immersed in PBS at 37℃, with the degradation property of the materials presented by the ratio of that remaining to the original amount of the freeze-dried hydrogels. **F** 3D reconstruction image of GelMA-MY-1-FITC hydrogel using confocal microscope. **G** FITC-labeled MY-1 was incorporated into GelMA and immersed in 10 mL PBS, with the peptide releasing speed calculated by the amount ratio of FITC-MY-1 in PBS over MY-1-FITC in total at a certain timepoint. Data expressed as mean ± SD. The experiments were repeated three times with similar results. ***, *p* < 0.001. Gel, GelMA; Gel-MY, GelMA-MY-1. Scale bars: 50 μm (in **A**), 20 μm (in **F**)

To investigate the effects of MY-1 on re-epithelialization in-vivo, MY-1 was incorporated into the GelMA hydrogels (GelMA-MY-1). Before employment in wound administration, the physicochemical properties of the GelMA and GelMA-MY-1 hydrogels were firstly characterized. The microstructures were examined by SEM, which revealed the similar porous appearance of both hydrogels (Fig. 3A).The hydrogels’ chemical elements were analyzed by EDS spectrum, with increased elements of N and O in GelMA-MY-1 noted, thus confirming the successful embedding of the MY-1 peptide into the GelMA (Fig. 3B).Then, Fourier transform infrared spectroscopy (FTIR) was carried out to detect whether the functional groups were changed after the incorporation of MY-1.The results revealed that the classic peak positions at 1540 cm^−1^ (N–H bending, amide II), 1646 cm^−1^ (C = O stretching, amide I), and 3400 cm^−1^ (N–H stretching, amide A) did not change following the incorporation of MY-1 (Fig. 3C), which indicated that the bioactive additive of GelMA had not changed during the incorporation process.

The swelling properties and degradation characteristics of the hydrogels revealed that both GelMA and GelMA-MY-1 reached swelling equilibrium after 12 h in PBS. The swelling ratio of GelMA-MY-1 (2408.86%) was slightly higher than that of GelMA (2245.37%), but did not show any statistical significance (*p* > 0.05) (Fig. 3D). Under comparison, GelMA and GelMA-MY-1 displayed similar degradability, with 50% of the weight remaining at day 10 and none remaining at day 14 (Fig. 3E). Collectively, GelMA and GelMA-MY-1 exhibited similar swelling and degradation characteristics, indicating that the incorporation of MY-1 did not significantly change the physical properties of GelMA.

To detect the peptide releasing capacity, MY-1 was first labeled with FITC (MY-FITC) and incorporated into the hydrogels (GelMA-MY-FITC). By confocal microscopic investigation and 3D reconstruction, the FITC-labeled MY-1 was evenly dispersed in GelMA hydrogel, suggesting the effective distribution of MY-1in the scaffold (Fig. 3F). The peptide releasing tests showed that nearly 60% of the GelMA-MY-1-FITC was released in the first 4 days, and gradually reached a releasing balance from then onward (Fig. 3G). Thus, GelMA successfully controlled the local releasing of MY-1.

### MY-1 accelerated skin wound closure through promoting re-epithelialization

**Fig. 4:**
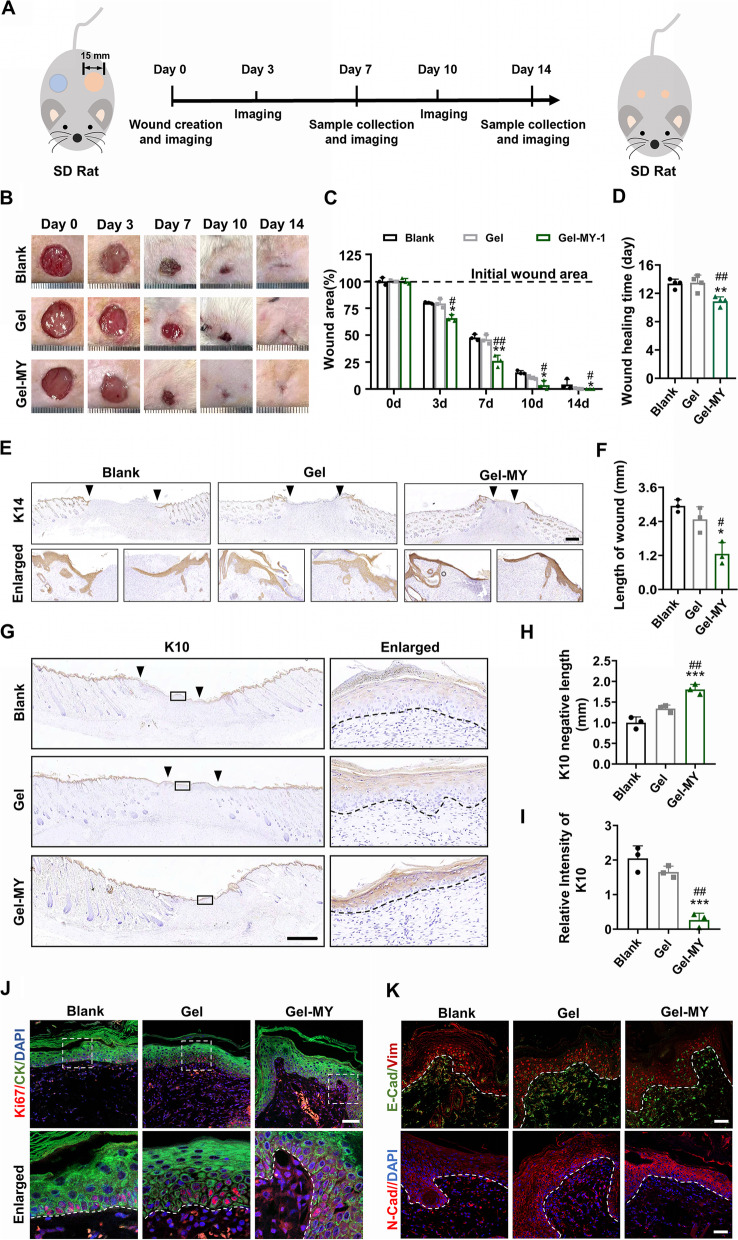
Locally released MY-1 accelerates wound re-epithelialization through promoting the migration and EMT of keratinocytes. **A** Schematic illustration of animal experiment design in SD rat wound healing models. **B** and **C** Full-thickness skin wounds (diameter: 15 mm) generated on the back of SD rats. 50 μL of GelMA hydrogel, GelMA-MY-1 hydrogel, and saline (Blank) applied, and the wounds covered with sterilized dressing. Wound images captured at days 0, 3, 7, 10, and 14 (**B**), and wound area measured and calculated at each timepoint (**C**). **D** Quantitation of the wound healing time in the three groups. **E** and** F** Immunohistochemical staining of K14 (**E**) carried out on wound samples harvested at day 7 post-surgery and the remaining wound width in each group (**F**). **G–I** Immunohistochemical staining of K10 (**G**) carried out on wound samples harvested at day 14 post-surgery (**G**), and the K10 negative distance (**H**), and intensity of K10 (**I**). **J** Immunofluorescence staining of Ki67 and pan-Cytokeratin on wound samples at day 7 post-surgery. **K** Immunofluorescence staining of E-cad, Vim, and N-cad in wound sections day 7 post-surgery. The data represent mean ± SD. *, *p* < 0.05; **, *p* < 0.01; ***, *p* < 0.001 vs the Blank group. #, *p* < 0.05; ##, *p* < 0.01; ###, *p* < 0.01vs the GelMA group. Scale bar: 3 mm (in **A**), 1 mm (in **D** and **F**), 100um (in **I**), 50um (in **J**). Abbreviation: Gel, GelMA; Gel-MY, GelMA-MY-1; K14, Cytokeratin 14; K10, Cytokeratin 10; CK, pan-Cytokeratin. E-cad, E-cadherin; Vim, Vimentin; N-cad, N-cadherin

After characterization, MY-1-loaded hydrogel was locally applied on the cutaneous wounds created on the back of the rat models (Fig. 4A). Analysis of the wound closure speed in the three groups indicated that GelMA-MY-1 markedly accelerated the wound healing process when compared to the wound areas in the GelMA group or the Blank group on the same timepoints (Fig. 4B). The wound coverage rate reached 74.0% at day 7 and 96.2% at day 10 in the GelMA-MY-1 group, compared to 52.2% and 84.6% in the Blank group, and 54.1% and 89.5% in the GelMA group at the respective timepoints (Fig. 4C). Regarding the wound healing duration, it took 10.9 days for the rats in the GelMA-MY-1 group to achieve complete wound coverage, and thus a much shorter duration than that of the GelMA group (13.5 day) and the Blank group (13.4 days) (Fig. 4D). In addition, immunohistochemistry (IHC) staining of cytokeratin 14 (K14), a special marker of basal keratinocytes, revealed that 7 days of MY-1 administration induced a longer epithelial tongue and a narrower wound gap (Fig. 4E and F). This result indicated that MY-1 might promote the re-epithelialization process via enhancing keratinocyte migration. On day 14, a spinous keratinocytes marker, cytokeratin 10 (K10), was used to stain the tissue sections harvested from the three groups, to facilitate a comparison of the differentiation and maturation speed of keratinocytes. The results of the IHC staining showed that MY-1 treatment led to a more mature spinous layer in the wound areas, which were composed of K10 + keratinocytes (Fig. 4G–I).

Besides migration, we next evaluated whether locally applied MY-1 promoted keratinocyte proliferation through detecting Ki67 expression in wound samples with immunofluorescence (IF) staining. In line with the MY-1-treated HaCaT cells, the results from the wound sections demonstrated that Ki67 was strongly expressed in the base of the epidermal layer, but failed to show any difference in Ki67 + keratinocyte densities in the three groups, suggesting that MY-1 might not directedly promote keratinocyte proliferation (Fig. 4J). To determine whether migrating keratinocytes acquired mesenchymal cell characteristics, we next detected the expression of classic EMT markers (E-cad, N-cad, and Vim) in the three groups. IF staining revealed that the MY-1-treated epidermis expressed lower content of E-cad and higher content of Vim, confirming that MY-1 enhanced wound re-epithelialization via promoting the EMT process of keratinocytes (Fig. 4K). Collectively, and consistent with the in-vitro results, we demonstrated that the local delivery of MY-1 accelerated skin wound re-epithelialization through enhancing keratinocytes’ EMT and migration.

### MY-1-induced keratinocyte migration and EMT via PI3K/AKT activation

**Fig. 5:**
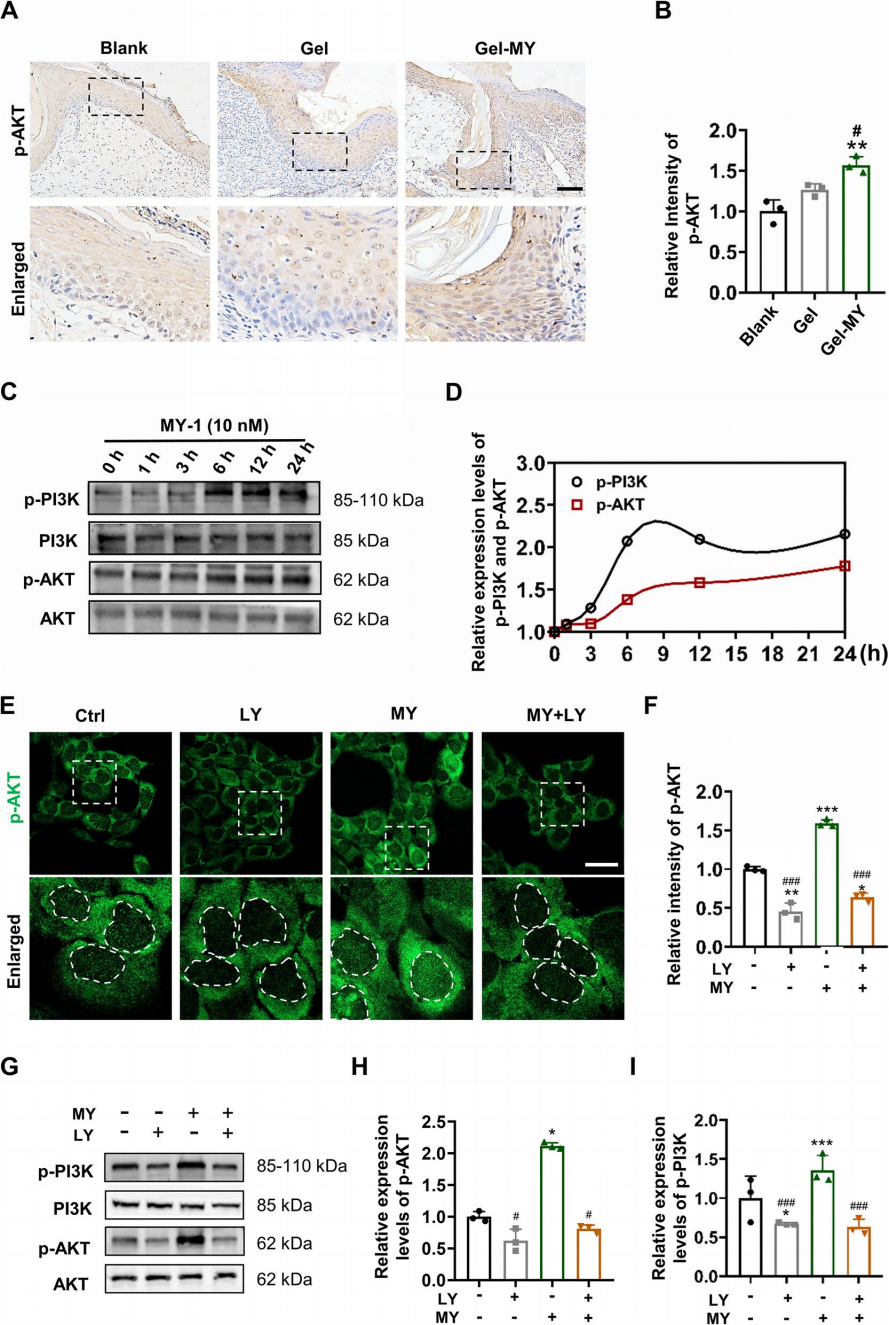
MY-1 activated PI3K/AKT signaling in wound areas and HaCaT cells. **A** and** B** Immunohistochemical staining of p-AKT carried out on wound samples harvested at day 7 post-surgery (**A**), and the intensity of p-AKT in three groups quantified and compared with the Blank (**B**). **, *p* < 0.01 vs Blank group; #, *p* < 0.05vs Gel group. **C** and** D** Time course assay carried out on HaCaT cells after treatment with MY-1 (10 nM) for different times, to measure the changes in the phosphorylation levels of PI3K and AKT (**C**). The quantified intensity of p-PI3K and p-AKT at each timepoint (**D**). **E** and **F** Immunofluorescence staining of p-AKT on HaCaT cells after subjecting to the different treatments (Control, LY 10 μM, MY 10 nM, MY + LY) for 6 h (**E**), and the intensity of p-AKT (**F**). **G–I** Western blot assay carried out to analyze the expression levels of p-PI3K and p-AKT on HaCaT cells treated with different mediums (**G**). The quantified ratios of p-PI3K/total PI3K and p-AKT/total AKT (**H** and **I**). The data represent mean ± SD. *, *p* < 0.05; **, *p* < 0.01; ***, *p* < 0.001 vs control group. #, *p* < 0.05; ##, *p* < 0.01 vs MY-1 group. Scale bar: 100 μm (in **A**),50 μm (in **E**). Gel, GelMA; Gel-MY, GelMA-MY-1

PI3K/AKT signaling is widely reported to play a critical role in cell polarization and migration in certain cell types. Our previous study reported that PI3K/AKT signaling is the main signaling activated in MY-1-treated dermal fibroblasts, but whether it exerts effects on keratinocytes remains unclear. To determine the role of PI3K/AKT signaling in MY-1-treated keratinocytes, we first evaluated the expression levels of p-AKT in wound samples. The IHC staining of p-AKT revealed that MY-1 administration increased the phosphorylation levels of AKT, which implied that MY-1 activated the PI3K/AKT signaling in keratinocytes (Fig. 5A and B). In line with the in-vivo results, the time-course experiments on HaCaT cells showed that an MY-1 dependent phosphorylation of PI3K and AKT began at 6 h after co-culturing with MY-1 (Fig. 5C and D). Moreover, to confirm the effects of PI3K/AKT signaling on MY-1-treated HaCaT cells, a specific PI3K inhibitor, LY, was employed to inhibit the activation of PI3K/AKT signaling. Immunofluorescence staining of p-AKT on the HaCaT cells revealed significantly attenuated phosphorylation levels of AKT and its nuclear translocation when the LY was presented (Fig. 5E and F). Similarly, western blotting analysis revealed that LY significantly inhibited the phosphorylation of PI3K and AKT (Fig. 5G-I). Collectively, these results demonstrated that PI3K/AKT signaling was activated in HaCaT cells following MY-1 administration.

**Fig. 6:**
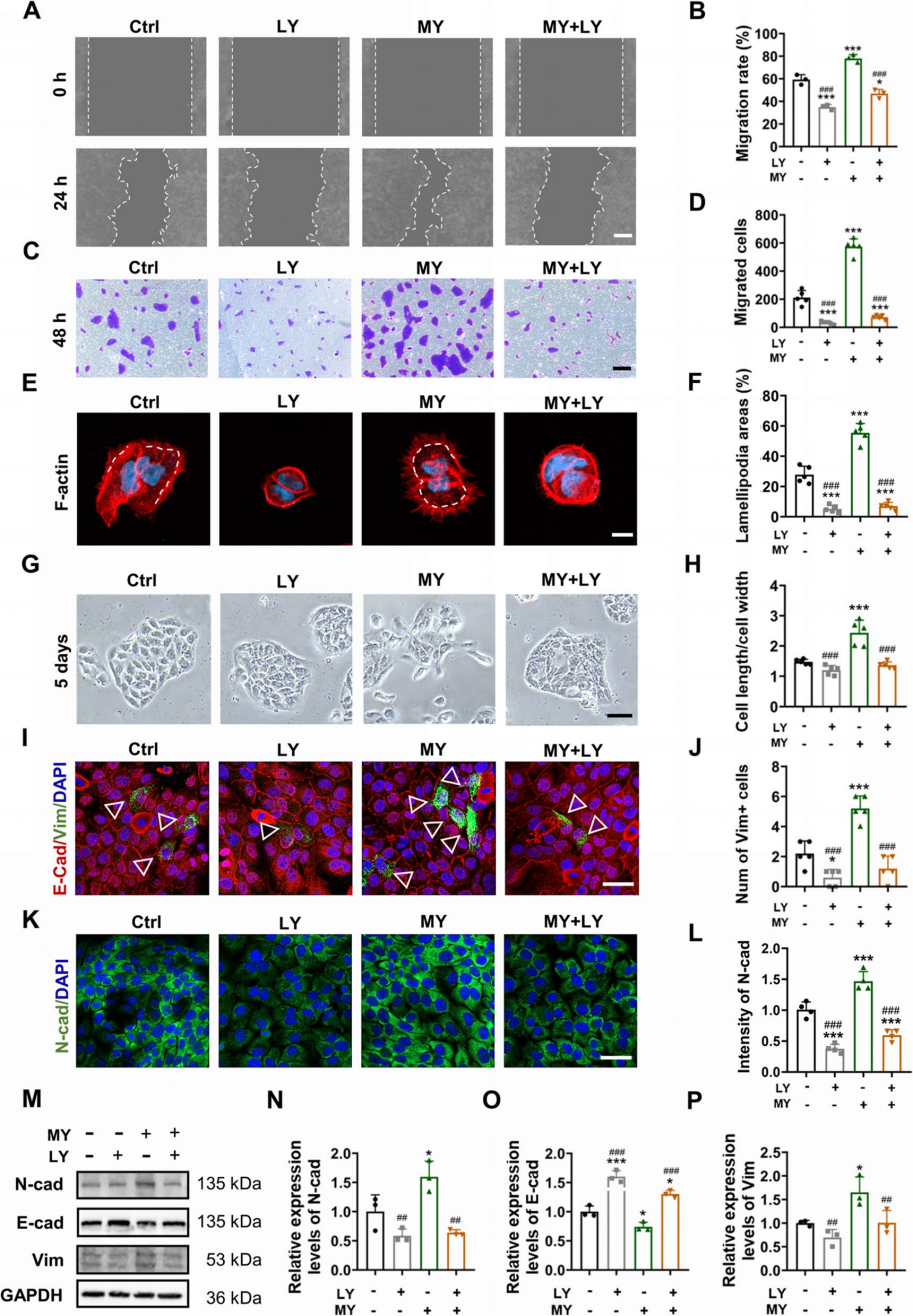
MY-1 accelerates migration and EMT of HaCaT cells via PI3K/AKT signaling. **A** and **B** Wound scratching assay images captured at 0 h and 24 h after incubation with/without MY-1 (10 nM) and/or LY (10 μM) (**A**), the quantified ratio of migrated areas (**B**). **C** and **D** Transwell assay images of the migrated HaCaT cells after incubation in different mediums for 48 h (**C**). The number of migrated cells in each group (**D**). **E** and** F** HaCaT cells incubated with different mediums for 12 h and the F-actin staining with rhodamine-phalloidin (**E**). The ratio of lamellipodia areas versus total cell areas measured and calculated (**F**). **G** and** H** Representative images of cellmorphology changes after incubating in a low serum medium containing different mediums for 5 days (**G**), and the ratio of the length/width of cells (**H**). **I** and** J** E-cad and Vim immunofluorescent staining on HaCaT cells (**I**), and the numbers of Vim + cells (**J**). **K** and** L** Immunofluorescent staining of N-cad performed on HaCaT cells after culturing with different mediums for 48 h (**K**), and relative intensity of N-cad (**L**). **M–P** The expression levels of EMT markers (E-cad, Vim, and N-cad) in HaCaT cells detected using western blot (**M**), and the intensity of bands measured and normalized to GAPDH and then calculated as the ratio of the controls (**N**–**P**). The data represent mean ± SD. *, *p* < 0.05; **, *p* < 0.01; ***, *p* < 0.001 vs control group. #, *p* < 0.05; ##, *p* < 0.01; ###, *p* < 0.001 vs MY-1 group. Scale bar: 100 μm (in **A**, **C**, and **G**), 20 μm (in **E**), 50 μm (in **I** and **K**). Abbreviation: Ctrl, Control; LY, LY294002; MY, MY-1; MY + LY, MY-1 + LY294002; E-cad, E-cadherin; Vim, Vimentin; N-cad, N-cadherin

Then, the relationship between MY-1-induced cell migration and activated PI3K/AKT signaling was evaluated. Both the scratch wound healing assay and Transwell assay revealed that the cell migration enhanced by MY-1 was markedly inhibited by LY, which indicated that MY-1 regulated HaCaT cell migration through PI3K/AKT signaling activation (Fig. 6A–D). Moreover, the increased lamellipodia formation, a cell phenomenon that represent enhanced cell motility, was also suppressed by PI3K/AKT inhibition (Fig. 6E and F). Therefore, the results verified that MY-1 regulated cell migration through activating PI3K/AKT signaling.

Next, the relationship between the MY-1-induced EMT and PI3K/AKT signaling was investigated. In cell morphology assay, the spindle-like shape and decreased density of MY-1-induced cells was eliminated when co-cultured with LY (Fig. 6G and 6H). In addition, IF staining showed that increased mesenchymal phenotype (Vim and N-cad) on MY-1-treated HaCaT cells was attenuated after PI3K/AKT signaling inhibition (Fig. 6I and L) which was subsequently confirmed by detecting the protein expression using western blot analysis (Fig. 6M–P) Therefore, we demonstrated that MY-1 induced keratinocyte migration and EMT via activating PI3K/AKT singling.

### Inhibition of PI3K/AKT signaling attenuated MY-1-enhanced wound re-epithelialization

**Fig. 7:**
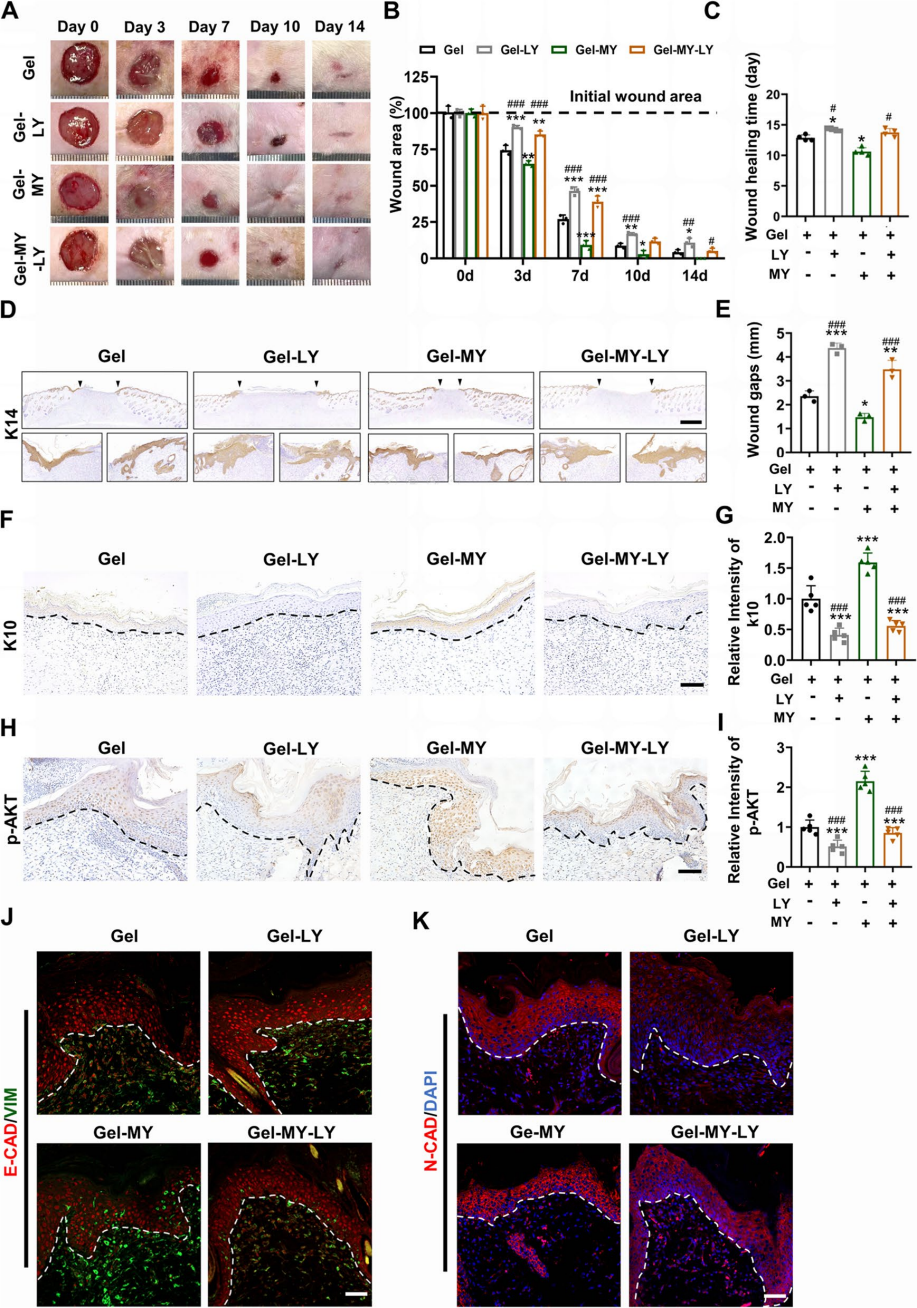
Inhibition of PI3K/AKT signaling attenuated wound re-epithelialization accelerated by MY-1. **A** and** B** Representative wound images from Gel, Gel-MY, Gel-MY and GelMA-MY-LY group captured at certain timepoints (**A**) and the wound area (%) at each timepoint analyzed and compared (**B**). **C** Quantitation and comparation of the wound healing time of four groups. **D** and** E** Immunohistochemical staining of K14 performed on wound samples harvested at day 7 post-surgery (**D**), and the remaining wound gaps in each group (**E**). **F** and** G** Immunohistochemical staining of K10 performed on wound samples harvested at day 14 post-surgery, and the intensity of K10 in each group (**G**). **H** and **I** Immunohistochemical staining of p-AKT performed on wound sections harvested at day 7 post-surgery (**H**). The intensity of p-AKT in each group (**I**). **J **Immunofluorescence staining of E-cad and Vim performed on wound samples harvested at day 7 post-surgery. **K** Immunofluorescence staining of N-cad performed on wound samples harvested at day 7 post-surgery. Data are expressed as mean ± SD of triple samples. *, *p* < 0.05; **, *p* < 0.01; ***, *p* < 0.001 vs Gel group. #, *p* < 0.05; ##, *p* < 0.01; ###, *p* < 0.001 vs Gel-MY group. Scale bars: 1 mm (in **D**), 100 μm (in **F** and **H**), 50 μm (in **J** and **K**). Abbreviation: Gel, GelMA; Gel-MY, GelMA-MY-1; Gel-MY-LY, GelMA-MY-1-LY294002; K14, Cytokeratin 14; K10, Cytokeratin 10

To determine whether the inhibition of the PI3K/AKT signaling reversed the MY-1-facilitated wound re-epithelialization, LY was employed on the wound models during MY-1 treatment. As expected, the addition of LY resulted in larger wound areas than solely MY-1 management, suggesting that the inhibition of PI3K/AKT signaling significantly delayed the wound healing process (Fig. 7A and B). Moreover, the presence of LY resulted to a longer wound healing duration when compared with those groups without LY (Fig. 7C). The IHC staining of K14 showed that the GelMA-MY-1 group exhibited the narrowest wound gaps, followed by the GelMA, Gel-MY-LY, and Gel-LY groups. These results suggested that co-treatment with LY could attenuate the acceleration effects of MY-1, demonstrating PI3K/AKT’s positive role in the MY-1-accelerated re-epithelialization process (Fig. 7D and E). Additionally, epithelial tongue formation was also notably disturbed when LY was administrated (Fig. 7D and E). On day 14, IHC staining on K10 revealed that blocking PI3K/AKT signaling also delayed the maturation of the newly covered keratinocytes (Fig. 7F and G). Phosphorylated AKT, the key participant of PI3K/AKT signaling in cell behavior regulation, was next detected in the four groups using IHC staining, with the aim of confirming that the PI3K/AKT signaling was successfully blocked. As per the results, the increased level of p-AKT was attenuated when co-treated with LY (Fig. 7H and I). Generally, these results demonstrated that the delayed re-epithelialization was closely related to the PI3K/AKT signaling inhibition.

Finally, the expressions of EMT markers in each group were evaluated, with the intention of detecting the effects of PI3K/AKT signaling on the MY-1-induced EMT process. IF staining of the wound samples revealed that the application of LY increased the expression of E-cad, while simultaneously inhibiting the expression of Vim and N-cad (Fig. 7J and K). Collectively, in this part we demonstrated that PI3K/AKT signaling inhibition suppressed the MY-1-enhanced wound re-epithelialization.

### MY-1 activated the PI3K/AKT signaling cascade via PTHR1

We next investigated the upstream signaling which activated PI3K/AKT signaling. Normally, PTH binds with PTH receptors (PTHR1) on the cell membranes and activates the downstream signaling pathways to regulate the cell behavior. Former study reported that activated PTH1R directly interacted with the subunit of PI3K (p85), thus triggering an intracellular signaling cascade to mediate cell activities [30]. Although MY-1 is an analog of PTH, the present results indicated that the effects of MY-1 and PTH on keratinocytes differ to a certain extent. Therefore, whether MY-1 activated PI3K/AKT signaling via PTHR1 remains unclear and is yet to be investigated.

**Fig. 8:**
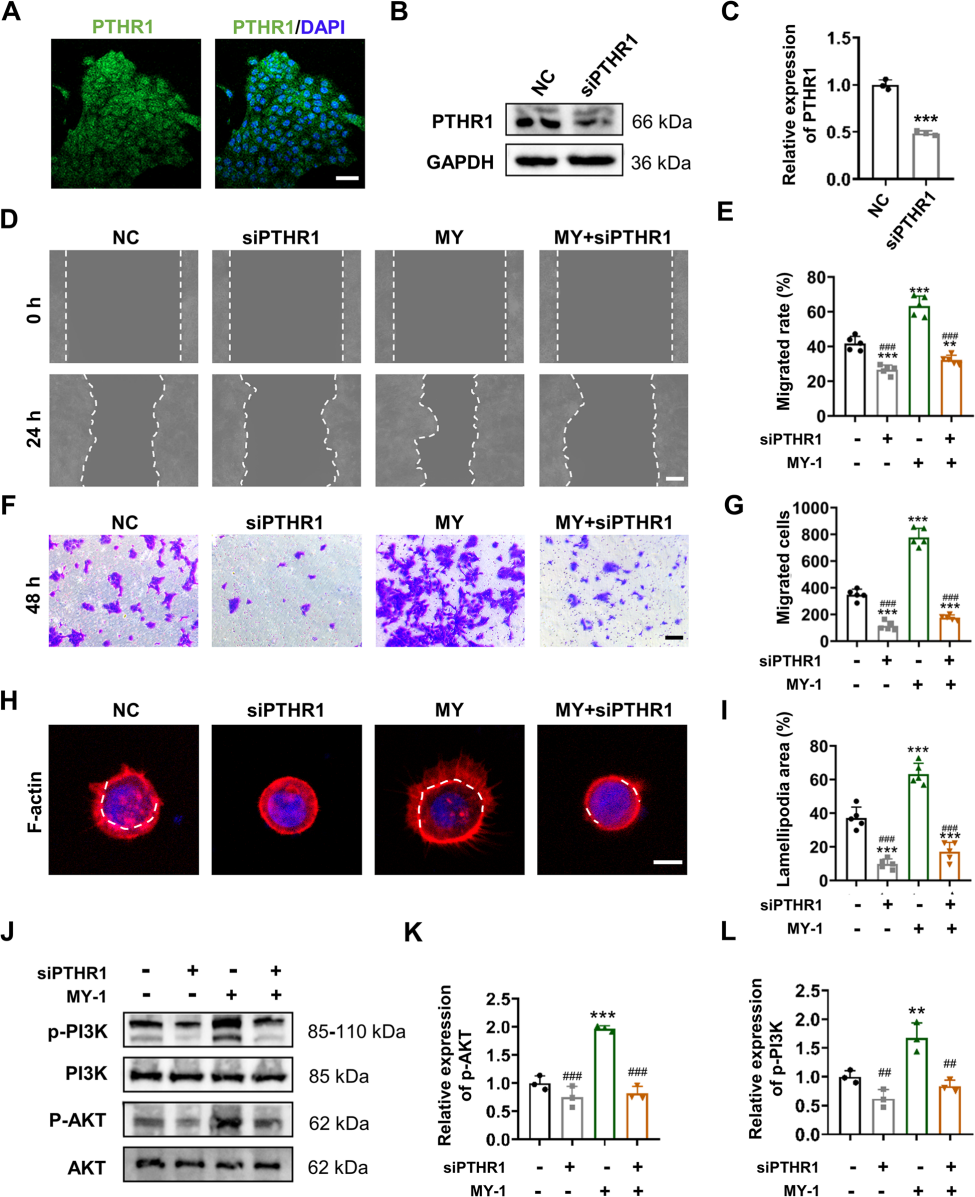
PTHR1 silencing blunted the activation effect of MY-1 on PI3K/AKT signaling and keratinocyte migration. **A** Immunofluorescent staining of PTHR1 on HaCaT cells. **B** and** C** Evaluation of the expression level of PTH1R in HaCaT cells after transfection with siRNA-PTHR1 (siPTHR1) using western blot analysis (**B**). The intensity of bands presenting protein expression level of PTHR1 (**C**). **D** and **E** HaCaT cells transfected with siPTHR1 or NC subjected to wound scratching assay. Images captured at 0 h and 24 h after incubation with/without MY-1 (**D**), and the quantified ratio of recovered areas over blank area at 0 h (**E**). **F** and **G** HaCaT cells transfection with siPTHR1 or NC pre-treated with MY-1 for 24 h and seeded in the upper chamber of Transwell plate. The Transwell images (**F**) and quantitative analysis (**G**) of the migrated HaCaT cells after incubation with/without MY-1 for 48 h.** H** and **I** HaCaT cells transfected with siPTHR1 or NC seeded in the confocal dishes and subjected to MY-1 for 12 h (**H**). The ratio of lamellipodia areas versus total cell areas (**I**). **J–L** Western blot assay carried out to evaluate the p-AKT and p-PI3K on siRNA-transfected HaCaT cells treated with PBS or MY-1 for 6 h (**J**). The ratios of p-PI3K/total PI3K and p-AKT/total AKT quantified and normalized to GAPDH, and then calculated as the ratio of the controls (**K** and **L**). The data represent mean ± SD. *, *p* < 0.05; **, *p* < 0.01; ***, *p* < 0.001 vs NC. #, *p* < 0.05; ##, *p* < 0.01; ###, *p* < 0.001 vs MY-1. Scale bars: 50 μm(in **A**), 100 μm (in **D** and **F**), 25 μm (in **H**). Abbreviation: NC, negative control; MY, MY-1

Previous studies reported that PTH receptors were widely expressed in diverse cell types (including keratinocytes) [20, 21], which were next confirmed in the present study by the immunofluorescent staining of PTHR1 (Fig. 8A). We then evaluated whether MY-1 regulates keratinocyte migration via PTHR1. HaCaT cells were transfected with specific siRNA targeting PTHR1, and the interference efficiency was evaluated by detecting the protein expression levels of PTHR1 using western blot analysis (Fig. 8B and C). After successful PTHR1 interference, both the wound scratching assay and transwell assay revealed that the cell migration enhanced by MY-1 was attenuated (Fig. 8D–G). Moreover, MY-1-induced lamellipodia formation was also inhibited with PTHR1 interference (Fig. 8H and I). Next, the relationship between activated PTHR1 and the PI3K/AKT signaling was detected. As shown in Fig. 8J–L, western blot analysis revealed that the phosphorylation of AKT and PI3K was disrupted following PTHR1 interference, which indicated that activated PTHR1 was the upstream of PI3K/AKT signaling. Therefore, we demonstrated that MY-1 regulated HaCaT cells’ migration through activating PTHR1-PI3K/AKT signaling.

## Discussion

In the present study, we designed a novel PTH analog named MY-1 and evaluated its role in keratinocyte migration and re-epithelialization. Using a classic drug carrier, we developed a local drug delivery system named GelMA-MY-1 and applied it on a rat skin wound model, aiming to evaluate MY-1’s impact on cutaneous wound healing, with particular focus on the process of re-epithelialization. The results of our studies revealed that the local application of MY-1 successfully accomplished skin wound re-coverage within a shorter duration, which was largely attributed to the accelerated re-epithelialization process induced by MY-1. Further study on epidermis and HaCaT cell lines revealed that MY-1 enhanced re-epithelialization through triggering the EMT and promoting the motility of keratinocytes, as opposed to stimulating their proliferation. Mechanistically, MY-1 regulates keratinocyte migration via PTHR1 and subsequently activating the downstream signaling of PI3K/AKT.

Wound re-epithelialization is an essential process for wound healing and is largely based on the proliferation, migration, and differentiation of keratinocytes. In our study, the IHC staining of K14 and K10 revealed that significantly more keratinocytes re-epithelializated the wound area after the locally administration of MY-1, thus encouraging us to evaluate MY-1’s role in the proliferation and migration of keratinocytes. Both the in-vivo and in-vitro studies suggested that the MY-1-enhanced wound re-epithelialization relied on promoting keratinocyte migration, rather than increasing the proliferation of keratinocytes. The null effect of MY-1 on cell proliferation was also identified in our previous studies, in which the proliferation of BMSCs [28], vascular endothelial cells, and fibroblasts was unchanged by MY-1. It appeared that MY-1 differed from PTH regarding the regulation of proliferation. *Yao *et al.revealed that PTH promoted wound coverage through multiple functions including, but not limited to inducing keratinocyte proliferation and migration [6]. The reason for the difference might be that the signaling, which is highly selectively initiated by MY-1, was not related to proliferation regulation. For example, after truncating the first two amino acids PTH (1–2), MY-1 lost the capacity to activate PLC and cAMP/PKA signaling but retained its role in osteogenesis and collagen deposition via PKC activation [26]; however, PTH(1–34) could activate multiple signaling including the above-mentioned pathways. Therefore, this structural change may also lead to the loss of PTH-induced cell proliferation. The definitive role and underlying mechanisms of MY-1in cell proliferation are yet to be reported.

EMT represents a transition of cells from sedentary, epithelial phenotype toward migratory, mesenchymal-like phenotype, a process that is indispensable for mammalians to re-establish intact epithelial coverage during wound healing [31]. Besides enhancing keratinocyte migration, MY-1 was found to have a pro-EMT function. MY-1 remarkably decreased the levels of E-cad in epidermis and HaCaT cells, while elevating mesenchymal markers including Vim and N-cad, thus demonstrating MY-1’s positive effect on the EMT of keratinocytes, which was coincident with PTH and related proteins to a certain degree. To date, PTH and related proteins have been identified as the effective EMT inductors in the other organs. For instance, *Guo *et al. pointed out that PTH could result in renal interstitial fibrosis via inducing EMT in tubular epithelial cells, while genistein ameliorated this situation by blocking PTH-induced α-SMA and connective tissue growth factor expression [24]. *He *et al. reported that PTH-like protein triggered EMT process in intestinal epithelial cells through binding PTHR1 and activating the downstream PKA/Runx2 axis [25]. *Ongkeko *et al. revealed that improved PTH-related protein (PTHrP) expression levels promoted an aggressive and metastatic phenotype in prostate cancer through inducing cells’ EMT, compared to a less motile phenotype when the PTHrP expression was knocked-down by siRNA [32]. How MY-1 and PTH peptides affect the EMT remains unknown, and the key genes that govern the EMT need to be explored.

The potential signaling activation by MY-1 in keratinocytes was then investigated. It was reported that numerous signaling pathways were involved in the re-epithelialization process, among which Wnt, TGF-β/Smads, and PI3K/AKT signaling were studied in depth [29, 33–36]. For PTH and analogs, however, the potential downstream signaling pathways by which they activated keratinocytes remain unclear. *Yao *et al. reported that the systemic administration of PTH promoted acute skin wound coverage through TGF-β/Smad3/mTOR pathways, while *Shen *et al. highlighted that their PTH derivative, PTHrP2, activated β-catenin and Akt/Erk1/2 signaling to accelerate skin wound healing process [6, 20]. Since both AKT and mTOR are the indispensable molecules of PI3K/AKT signaling, these studies implied the critical role of PI3K/AKT in PTH-regulated cells. As a classic pathway, PI3K/AKT is usually recognized as a downstream signaling of epidermal growth factor receptor or platelet-derived growth factor receptor, which modulates a variety of cell behaviors including metabolism, proliferation, differentiation, and migration via AKT phosphorylation and downstream genes’ activation [34]. Activated PI3K/AKT signaling was found to play a critical role in cell polarity and migration regulating, and thus is indispensable for the EMT and migration of keratinocytes [37, 38]. Based on the afore-mentioned studies and the previous RNA-sequence analysis on MY-1-stimulated fibroblasts (data not shown), we hypothesized that PI3K/AKT is one of the pathways activated by MY-1. Our present study revealed that the upregulated phosphorylation levels of PI3K and AKT were closely accompanied with enhanced keratinocyte migration and EMT following MY-1 treatment, whereas such promoted motility and EMT were subsequently inhibited by the selective PI3K/AKT signaling inhibitor LY294002. Hence, and consistent with former reported analogs, here we demonstrated that MY-1-induced keratinocytes’ EMT and migration were closely related to the activation of the PI3K/AKT pathway.

MY-1 is a newly designed PTH analog that exhibits unique functions from PTH by truncating and repeating a partial amino acid sequence of hPTH (1–34). To determine whether MY-1 still regulates cell behavior via PTHR1, we interfered with the PTHR1 expression in HaCaT cells through specific siRNA transfection. We found that the enhanced cell motility by MY-1 treatment was significantly suppressed, suggesting that PTHR1 remains one of the receptors targeted by MY-1. As a classic G protein-coupled receptor, PTHR1 mainly regulates cell activities through activating PKC/cAMP, PKC, and PLC signaling, while some studies also revealed that PTHR1 can activate Wnt and TGF-β signaling. The relationship between PTHR1 and PI3K signaling was explored in several studies. For example, *Yamamoto *et al. investigated this relationship and found that activated PTHR1 directly interacts with the regulatory subunit of PI3K (p85α) in a PTH-dependent manner, resulting in a PTHR1-PI3K/AKT-bad signaling cascade that prevents osteoblast-like cells from undergoing apoptosis [30]. Therefore, although co-immunoprecipitation experiments, gene ablation and rescue analysis or other binding investigations between MY-1 and PTHR1 have not been performed, the mediation effect of PTHR1 could be verified by the siRNA technique, and thus we believe that MY-1 activates PI3K/AKT signaling through PTHR1 signaling. The microstructural adaptation of ligand (MY-1 vs PTH) and receptor (PTHR1), as well as the possible downstream signaling effectors, will be extensively explored in future work.

## Conclusion

**Fig. 9:**
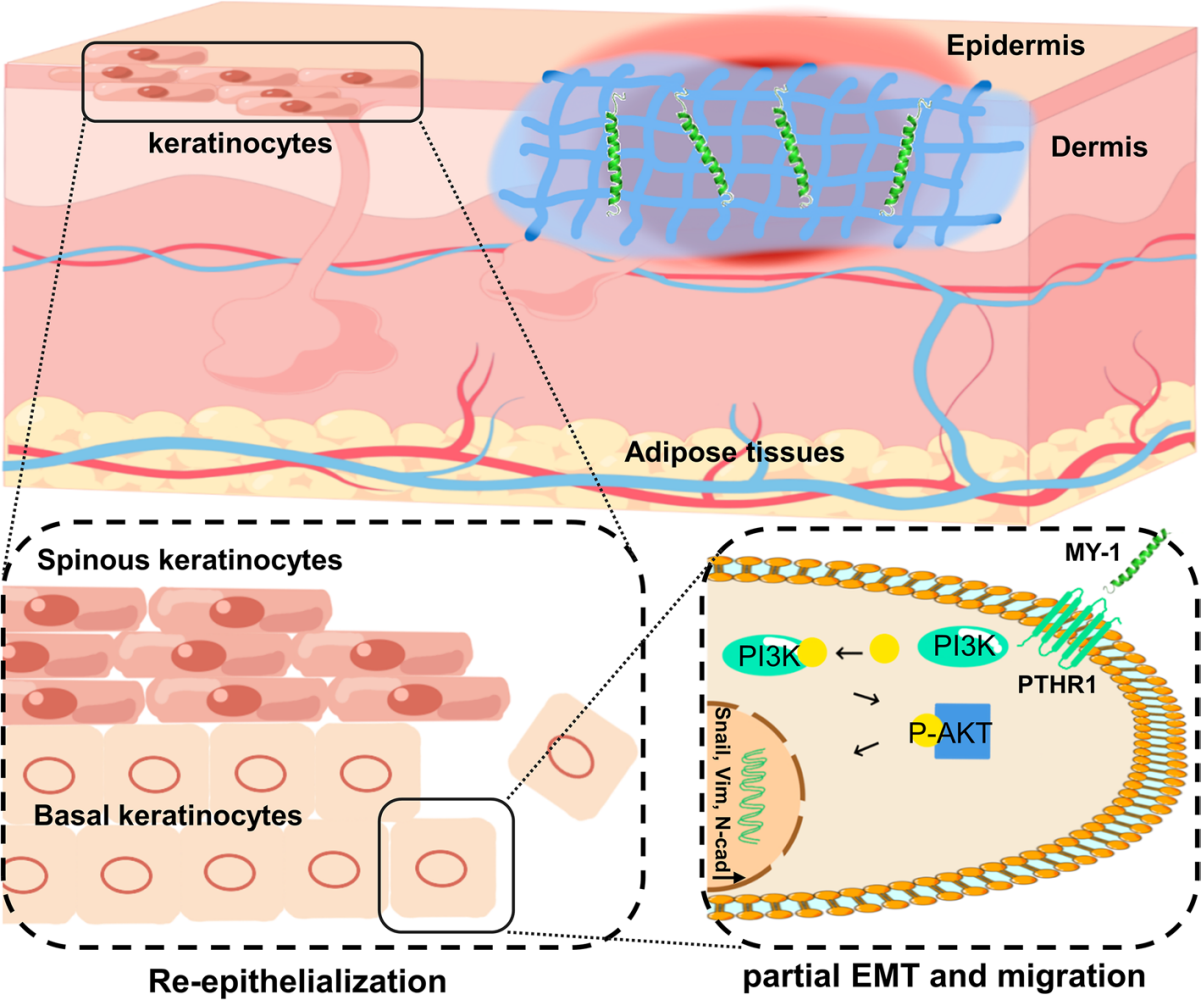
The mechanism diagram of how MY-1 promoted wound re-epithelialization. The released MY-1 from GelMA-MY-1 hydrogel binds with PTH1R and subsequently activates the PI3K/AKT signaling in the downstream to enhance keratinocytes’ EMT and migration. Enhanced motility of keratinocytes then results in an accelerated re-epithelialization process

In the present study, we demonstrated that a PTH analog (MY-1) promoted re-epithelialization through regulating keratinocyte migration and EMT via PTHR1-PI3K/AKT activation (Fig. 9), which provided a useful tool for studying the mechanism of cell migration in wound healing and a possible drug candidate for wound treatment.

## Data Availability

All data generated or analyzed during this study are included in this published article.

## Abbreviations

PTH: Parathyroid hormone
EMT: Epithelial-mesenchymal transition
p-PI3K: Phosphorylated PI3K
p-AKT: Phosphorylated AKT
PTHR1: Parathyroid hormone type I receptor
HaCaT: Human immortalized keratinocytes
TGF-β: Transforming growth factor beta
EGF: Epidermal growth factor
FGF: Fibroblast growth factor
TNF-α: Tumor necrosis factor-alpha
ECM: Extracellular matrix
GelMA: Gelatin methacryloyl
TFA: Trifluoroacetic acid
