# Correction: Human Parathyroid Hormone Analog (3–34/29–34) promotes wound re-epithelialization through inducing keratinocyte migration and epithelial–mesenchymal transition via PTHR1-PI3K/AKT activation

**DOI:** 10.1186/s12964-023-01318-7

**Published:** 2023-09-19

**Authors:** Chunhao Zhou, Donghua Guan, Jialiang Guo, Shangbo Niu, Zhihai Cai, Chengfu Li, Chenghe Qin, Wenjuan Yan, Dehong Yang

**Affiliations:** 1 Division of Spine Surgery, Department of Orthopaedics, Nanfang Hospital, Southern Medical University, 1838 North Guangzhou Ave, Guangzhou, 510515 P. R. China; 2 Department of Emergency, Zengcheng Branch of Nanfang Hospital, Southern Medical University, No. 28 Chuangxin Avenue Yongning Street, Guangzhou, 511340 P. R. China; 3 Division of Orthopaedic Trauma, Department of Orthopaedics, Nanfang Hospital, Southern Medical University, 1838 North Guangzhou Ave, Guangzhou, 510515 P. R. China; 4 Department of Stomatology, Nanfang Hospital, Southern Medical University, 1838 North Guangzhou Ave, Guangzhou, 510515 P. R. China

**Correction: Cell Commun Signal 21, 217 (2023)**

**https://doi.org/10.1186/s12964-023-01243-9**

Following publication of the original article [1], the authors wish to clarify their affiliations. The affilaitions in this correction article are displayed correctly (specifically affiliation 1 and 3).
